# Acute supplementation of *N*‐acetylcysteine does not affect muscle blood flow and oxygenation characteristics during handgrip exercise

**DOI:** 10.14814/phy2.12748

**Published:** 2016-04-06

**Authors:** Joshua R. Smith, Ryan M. Broxterman, Carl J. Ade, Kara K. Evans, Stephanie P. Kurti, Shane M. Hammer, Thomas J. Barstow, Craig A. Harms

**Affiliations:** ^1^ Department of KinesiologyKansas State UniversityManhattanKansas; ^2^ Department of Health and Exercise ScienceUniversity of OklahomaNormanOklahoma

**Keywords:** Blood flow, *N* ‐acetylcysteine, vasodilation

## Abstract

*N*‐acetylcysteine (NAC; antioxidant and thiol donor) supplementation has improved exercise performance and delayed fatigue, but the underlying mechanisms are unknown. One possibility is NAC supplementation increases limb blood flow during severe‐intensity exercise. The purpose was to determine if NAC supplementation affected exercising arm blood flow and muscle oxygenation characteristics. We hypothesized that NAC would lead to higher limb blood flow and lower muscle deoxygenation characteristics during severe‐intensity exercise. Eight healthy nonendurance trained men (21.8 ± 1.2 years) were recruited and completed two constant power handgrip exercise tests at 80% peak power until exhaustion. Subjects orally consumed either placebo (PLA) or NAC (70 mg/kg) 60 min prior to handgrip exercise. Immediately prior to exercise, venous blood samples were collected for determination of plasma redox balance. Brachial artery blood flow (BABF) was measured via Doppler ultrasound and flexor digitorum superficialis oxygenation characteristics were measured via near‐infrared spectroscopy. Following NAC supplementaiton, plasma cysteine (NAC: 47.2 ± 20.3 *μ*mol/L vs. PLA: 9.6 ± 1.2 *μ*mol/L; *P* = 0.001) and total cysteine (NAC: 156.2 ± 33.9 *μ*mol/L vs. PLA: 132.2 ± 16.3 *μ*mol/L; *P* = 0.048) increased. Time to exhaustion was not significantly different (*P* = 0.55) between NAC (473.0 ± 62.1 sec) and PLA (438.7 ± 58.1 sec). Resting BABF was not different (*P* = 0.79) with NAC (99.3 ± 31.1 mL/min) and PLA (108.3 ± 46.0 mL/min). BABF was not different (*P* = 0.42) during exercise or at end‐exercise (NAC: 413 ± 109 mL/min; PLA: 445 ± 147 mL/min). Deoxy‐[hemoglobin+myoglobin] and total‐[hemoglobin+myoglobin] were not significantly different (*P* = 0.73 and *P* = 0.54, respectively) at rest or during exercise between conditions. We conclude that acute NAC supplementation does not alter oxygen delivery during exercise in men.

## Introduction

The production of free radicals occurs at rest (Richardson et al. 2007; Bailey et al. 2007) and increases during exercise (Richardson et al. 2007; Bailey et al. 2003, 2004, 2007). Although endogenous antioxidant systems (e.g., superoxide dismutase, catalase, glutathione peroxidase) help minimize the increase in free radicals (Sen 1995), the accumulation of free radicals occurs with exercise (Richardson et al. 2007; Bailey et al. 2003, 2004, 2007), which affects processes associated with muscular fatigue (Reid 2015). In 1990, *N* ‐acetylcysteine (NAC), an antioxidant and thiol donor, was shown to delay fatigue in rabbit diaphragm fibers (Shindoh et al. 1990). Since then, NAC supplementation has been reported to delay fatigue and/or improve exercise performance with small muscle mass (Reid et al. 1994; Supinski et al. 1997; Travaline et al. 1997; Matuszczak et al. 2005; Kelly et al. 2009; Smith et al. 2014), and large muscle mass exercise (Corn and Barstow 2011; Medved et al. 2004b; McKenna et al. 2006; Slattery et al. 2014; Cobley et al. 2011). However, this is not a consistent finding in large muscle mass exercise (Medved et al. 2003, 2004a; Bailey et al. 2011; Trewin et al. 2013), with some studies reporting high intersubject variability (Bailey et al. 2011; Medved et al. 2004a). Although results may be conflicting, mechanistically, NAC has been reported to positively affect Na+‐K+ ATPase pump activity (McKenna et al. 2006) and regulation of calcium release within the myofibril (Andrade et al. 2001).

It has been speculated that NAC may have positive cardiovascular affects (Reid et al. 1994; Slattery et al. 2014; Reid 2015), which may also contribute to delay exercise‐induced fatigue. NAC has been suggested to augment the effects of nitric oxide (Simon et al. 1993) and increase flow‐mediated dilation using bovine vascular rings (Cooke et al. 1990). These data suggest NAC supplementation may lead to greater nitric oxide bioavailability and exercise‐induced vasodilation.

During low‐intensity exercise, free radicals contribute to vasodilation via vasoactive properties (Richardson et al. 2007; Liu et al. 2003). Therefore, it remains unlikely NAC supplementation would lead to increased blood flow during low‐intensity exercise. In support, NAC supplementation does not alter limb blood flow at rest (Smith et al. 2014; Nyberg et al. 2012) nor during moderate‐intensity knee extension exercise (Nyberg et al. 2012) despite reducing venous oxidized glutathione in healthy young men (Nyberg et al. 2014). In contrast, higher exercise intensities lead to greater free radical production (Bailey et al. 2004, 2003), which can attenuate nitric oxide (NO) bioavailability (Thomas et al. 2006) and exercise‐induced vasodilation. Therefore, NAC may lead to greater vasodilation and increased blood flow during severe‐intensity exercise. During severe‐intensity cycling exercise, NAC supplementation did not increase plasma nitrite levels compared to placebo (Bailey et al. 2011), but locomotor muscle blood flow was not directly measured. In this study, we chose small muscle mass exercise (i.e., handgrip) because previous studies have consistently found improvements in small muscle mass exercise performance with NAC (Ferreira and Reid 2008). We were also interested in determining if NAC influences muscle oxygenation characteristics because antioxidant supplementation has recently been shown to lower microvascular oxygenation at rest and during muscle contractions (Copp et al. 2009). It is currently unclear if NAC supplementation affects oxygen delivery during severe‐intensity exercise which could be a mechanism as to how NAC improves exercise performance. The purpose of the study, therefore, was to determine if NAC supplementation influenced exercising limb blood flow and muscle oxygenation characteristics during severe‐intensity handgrip exercise to fatigue in healthy men. We hypothesized that acute oral NAC supplementation would increase limb blood flow and lower muscle deoxygenation during severe‐intensity exercise.

## Methods

Eight healthy, nonendurance trained men were recruited as subjects and participated in this study (age: 21.8 ± 1.2 years; height: 174.9 ± 9.3 cm; weight: 77.1 ± 17.5 kg; peak power: 6.0 ± 1.3 W). Each subject provided written consent and medical health history prior to testing. All subjects were free of chronic and/or acute cardiovascular and metabolic diseases. Subjects refrained from antioxidant supplementation for at least 1 week prior to and over the course of the study. Subjects abstained from vigorous physical activity for 12 h and food and caffeine ingestion for 4 h prior to testing. All experimental procedures were approved by the Institutional Review Board at Kansas State University, Manhattan, KS and conformed to the Declaration of Helsinki.

### Experimental design

This study was a randomized double‐blind placebo controlled cross‐over design. Subjects reported to the laboratory on four different occasions. First, subjects performed an incremental handgrip test to determine peak power (P_peak_). On the second visit, subjects performed a familiarization trial of handgrip exercise at 80% P_peak_ to exhaustion. The third and fourth visits were randomized with a washout time of at least 1 week (Reid et al. 1994; Kelly et al. 2009). One hour prior to handgrip exercise, subjects orally consumed 70 mg/kg of either NAC or placebo (PLA) consisting of cellulose. One hour following NAC or PLA ingestion and immediately prior to handgrip exercise, a venous blood sample was taken from the antecubital vein for plasma redox analyses. Then, subjects performed handgrip exercise at 80% P_peak_ to exhaustion.

### NAC Supplementation

NAC and PLA (cellulose) pills (600 mg per pill) were used for oral administration. The NAC and PLA pills were similar in size, shape, and color. One investigator prepared the pills and performed the randomization for the entire study and was not present for data collection. Investigators and subjects were blinded to the pills during data collection. In this study, an acute oral dosage of 70 mg/kg of NAC and PLA was consumed 1 hr prior to handgrip exercise. This NAC dosage was used because it was reported to increase plasma total cysteine, cysteine, and decrease oxidized glutathione with no adverse reactions (Ferreira et al. 2011). Because whole pills were administered for each subject, the number of pills administered was matched to equal 70 mg/kg as closely as possible. Consumption of NAC supplementation 1 hr prior was used because plasma NAC and cysteine have been reported to peak within 60–120 min of ingestion (Borgstrom and Kagedal 1990).

### Handgrip ergometer

A custom built handgrip ergometer was used for all exercise testing as previously described (Broxterman et al. 2014). When the subjects were seated at the handgrip ergometer and placed their hands on the handrail, their forearms were at heart level and elbows were slightly bent. The contraction frequency was 20 contractions per min with a 50% duty cycle. Correct timing was ensured with an audio recording set with the specific timing of the duty cycle and feedback provided by the investigator monitoring the tests. For the incremental handgrip test, the test started at 1.0 W and power output was increased 0.5 W each minute until exhaustion. The P_peak_ was the highest power output attained by the subjects for which at least 30 sec of the stage was completed. For the submaximal handgrip tests, subjects completed constant power (80% P_peak_) tests to exhaustion. This handgrip protocol (contraction frequency, duty cycle, and intensity) has recently been shown to lead to the development of peripheral muscle fatigue (Broxterman et al. 2015).

### Doppler ultrasound

Brachial artery blood velocity was measured via Doppler ultrasound (Vivid 3; GE Medical Systems, Milwaukee, WI). The gate of the Doppler was set to full width of the brachial artery to ensure complete insonation. All Doppler velocity measurements were corrected for the angle of insonation, which was adjusted to be less than 60 degrees. Measurements in the brachial artery were made 2–5 cm above the antecubital fossa. Mean blood velocity (${\dot{V}}_{\mathrm{mean}}$; cm × sec^−1^) was defined as time averaged mean velocity over each 3 s contraction cycle. All (${\dot{V}}_{\mathrm{mean}}$ were determined over the last three contraction cycles of each min and at end‐exercise. Brachial artery blood flow (BABF) was calculated as the product of ${\dot{V}}_{\mathrm{mean}}$ and vessel cross‐sectional area (CSA). Brachial artery diameters were measured in the transverse axis using two‐dimensional sonography and used to calculate vessel CSA (CSA = Πr^2^; cm^2^). Brachial artery diameters were measured at rest and each min of exercise. Brachial artery diameters were measured by the same investigator who was blinded to the condition. Shear rate was defined as 4·(${\dot{V}}_{\mathrm{mean}}D^{-1}$), where D is diameter in centimeters.

### Near‐infrared spectroscopy

A frequency‐domain multi‐distance Near‐infrared spectroscopy (NIRS) system (Oxiplex TS, ISS, Champaign, IL) was used to determine the oxygenation characteristics of the flexor digitorum superficialis. Briefly, this NIRS device consists of eight light‐emitting diodes (LED) operating at wavelengths of 690 and 830 nm with four LEDs per wavelength and with one detector fiber bundle and LED‐detector separation distances of 2.0, 2.5, 3.0, and 3.5 cm. Electromyography and palpation was used to locate the flexor digitorum superficialis on the right arm and the NIRS probe was securely placed longitudinally along the belly of the muscle. Next, the probe placement was marked with indelible ink for reproducible placement. The NIRS probe was calibrated prior to each test using a calibration block with known scattering coefficients and absorption and calibration was confirmed on a separate block with different absorption and scattering coefficients. The NIRS data were collected at 50 Hz and stored for post hoc analysis. The NIRS data were processed using 3 s averages and three consecutive contraction cycles at the end of each minute were averaged. The deoxy‐[hemoglobin+myoglobin] (deoxy‐[Hb+Mb]) is relatively insensitive to changes in blood volume (De Blasi et al. 1993; Ferrari et al. 1997; Grassi et al. 2003) and has been used to estimate fractional oxygen extraction (De Blasi et al. 1994; Ferrari et al. 1997; Grassi et al. 2003). The NRIS device used provides absolute concentrations (*μ*mol/L) for deoxy‐[Hb+Mb] and oxygenated‐[hemoglobin+myoglobin] (oxygenated‐[Hb+Mb]), which sum to determine total‐[hemoglobin+myoglobin] (total‐[Hb+Mb]), as well as a tissue oxygenation index (TOI; %Saturation of [hemoglobin+myglobin]). Dynamic reduced scattering coefficients were measured and incorporated in all of the NIRS data calculations.

### Blood analysis

One hr following the acute oral consumption of NAC and PLA 2–5 mL of venous blood were slowly drawn from the antecubital vein. If the samples were hemolyzed, they were discarded and an additional venous blood draw was performed. The venous blood sample was added to a microcentrifuge tube containing a preservative solution (2 mg iodoacetic acid, 1 mg bathophenanthroline disulfonate, and 100 mmol/L serine‐borate (pH 8.5) containing 0.5 mg sodium heparin) and inverted gently. The microcentrifuge tube was centrifuged (29,905 *g*, 30 sec) and then 200 *μ*L of supernatant was added to a tube containing 200 *μ*L of perchloric acid solution and stored at −60°C for later analysis. High performance liquid chromatography was used to analyze the plasma samples for glutathione (GSH), oxidized glutathione (GSSG), cysteine (CySH), cystine (CySS), and cysteine‐glutathione disulfide (CySSG) (Clinical Biomarkers Laboratory, Emory University, Atlanta, GA) as previously done (Ferreira et al. 2011). Total glutathione (tGSH) was calculated as GSH + 2**·**GSSG + CySSG. Total cysteine (tCyS) was calculated as CySH + 2·CySS + CySSG.

### Statistical analysis

Sigma Statistical (Jandel Scientific Software, Chicago, IL) was used for data analysis. Data are presented as mean ± SD. Normality of the data was confirmed using Shapiro–Wilk normality tests. Paired *t*‐tests were used to determine differences in time to exhaustion, preexercise plasma redox values, and absolute changes in diameter, blood flow and shear rate (end‐exercise minus resting) between conditions. Differences in cardiovascular variables and muscle oxygenation characteristics were determined by a 2 × 2 (group × time) repeated measures analysis of variance (ANOVA) and the F statistics (*F*) were reported. A Tukey's post hoc analysis was performed to determine where significant differences existed. Statistical significance was set at *P* < 0.05 for all analyses.

## Results

### NAC supplementation

**Figure 1:**
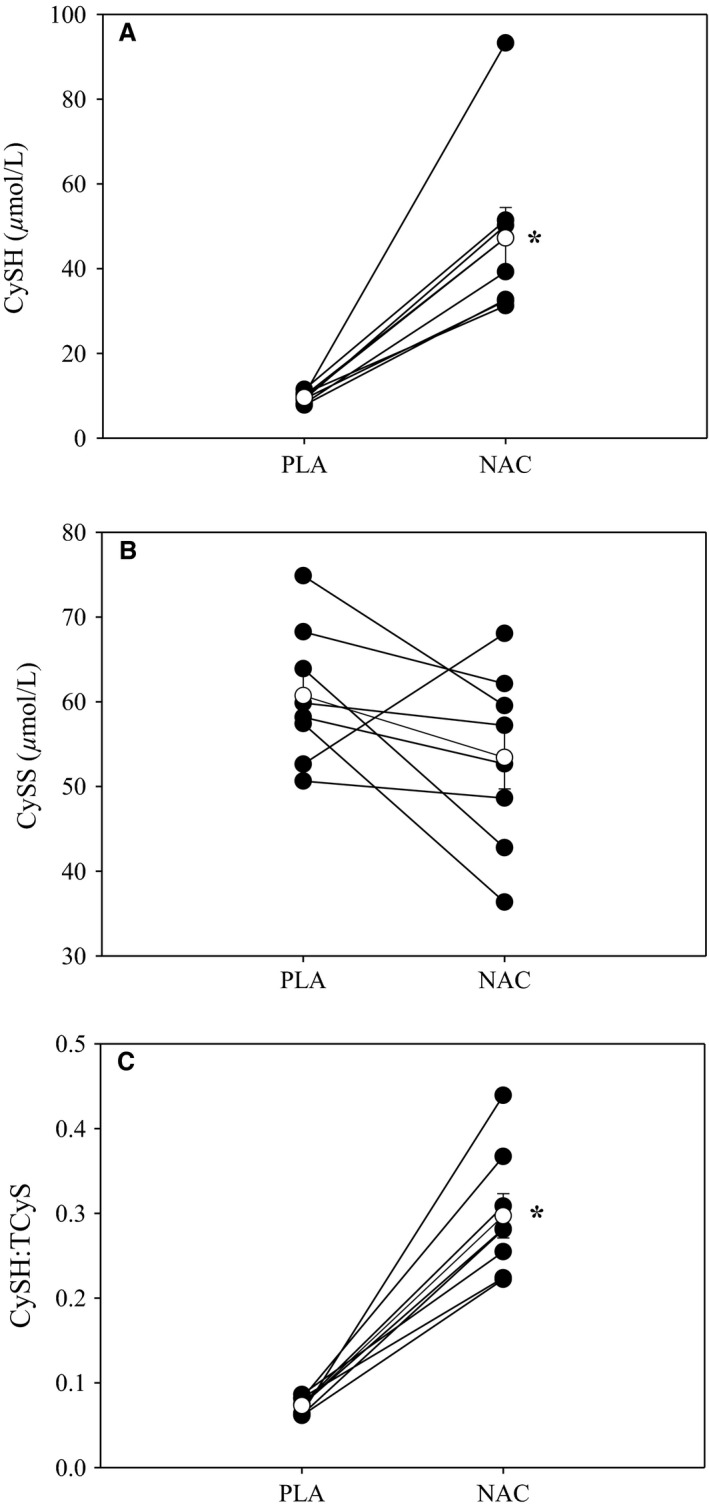
Plasma cysteine derivatives with NAC and PLA. Individual (closed circles) and mean (open circles) preexercise plasma CySH (A), CySS (B), and CySH:tCyS (C) following NAC and PLA supplementation. NAC supplementation led to significant increases CySH and CySH:tCyS. NAC, *N*‐acetylcysteine;pla, placebo;CySH, cysteine; CySS, Cystine; tCyS, Total cysteine; tGSH, Total glutathione.

The NAC group orally consumed ~67 mg/kg and reported no gastrointestinal distress. Figure 1 shows preexercise CySH, CySS, and CySH:tCyS following NAC and PLA supplementation. Following NAC supplementation, CySH (NAC: 47.2 ± 20.3 *μ*mol/L vs. PLA: 9.6 ± 1.2 *μ*mol/L; *P* = 0.001), tCyS (NAC: 156.2 ± 33.9 *μ*mol/L vs. PLA: 132.2 ± 16.3 *μ*mol/L; *P* = 0.048), GSSG (NAC: 0.18 ± 0.08 *μ*mol/L vs. PLA: 0.04 ± 0.02 *μ*mol/L; *P* < 0.001), tGSH (NAC: 6.2 ± 1.0 *μ*mol/L vs. PLA: 4.7 ± 1.4 *μ*mol/L; *P* = 0.027), and CySH/tCyS (NAC: 0.30 ± 0.07 *μ*mol/L vs. PLA: 0.07 ± 0.01 *μ*mol/L; *P* < 0.001) were significantly higher and GSH/GSSG was lower (NAC: 22.9 ± 9.2 *μ*mol/L vs. PLA: 76.8 ± 17.6 *μ*mol/L; *P* < 0.001) compared to PLA. Following NAC supplementation, the increased CySH, GSSG, and CySH:tCyS occurred in all eight subjects, while the increases in tCyS and tGSH occurred in six of eight subjects.

### Time to exhaustion

**Figure 2:**
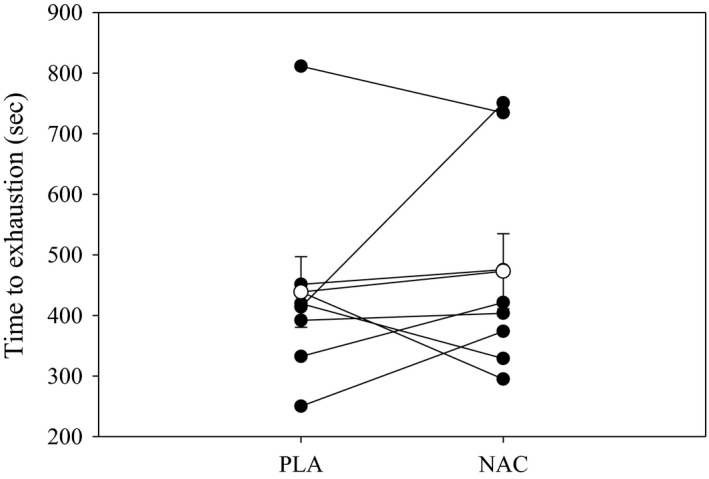
Time to exhaustion with NAC and PLA. Mean (open circles) and individual (closed circles) time to exhaustion values for NAC and PLA supplementation. There were no significant differences in time to exhaustion between conditions. NAC, *N*‐acetylcysteine;pla, placebo.

Figure 2 shows the mean and individual time to exhaustion for NAC and PLA. Time to exhaustion was not different between NAC and PLA (NAC: 473.0 ± 62.1 sec vs. PLA: 438.7 ± 58.1 sec; *P* = 0.55). There was inherent intersubject variability with five subjects showing improved performance (+3%, +49%, +27%, +81%, +5%) and three subjects showing decreased performance (−21%, −9%, −33%). No significant associations existed between the percent changes in plasma redox balance markers and exercise performance.

### Brachial artery blood flow

**Figure 3:**
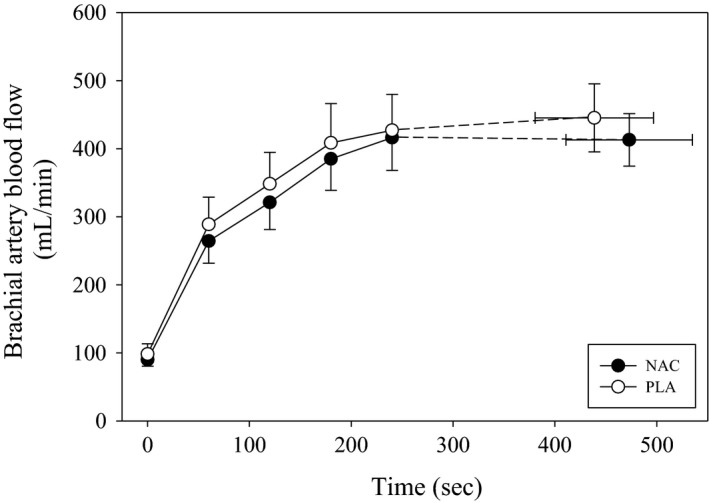
Mean blood flow for NAC and PLA during exercise. Mean BABF at rest and during exercise for NAC (closed circles) and PLA (open circles) supplementation. BABF increased (*P* < 0.05) from rest to exercise in both conditions. There were no significant differences between NAC and PLA. NAC, *N*‐acetylcysteine; pla, placebo; BABF, brachial artery blood flow

**Figure 4:**
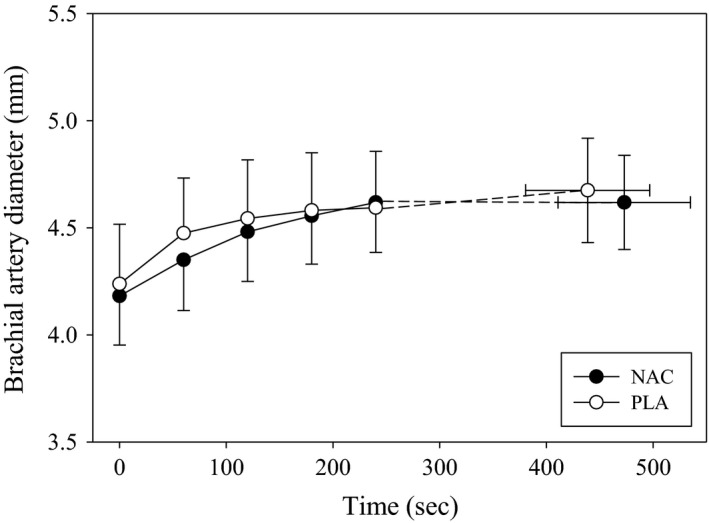
Mean brachial artery diameters for NAC and PLA during exercise. Mean brachial artery diameter at rest and during exercise for NAC (closed circles) and PLA (open circles) supplementation. Brachial artery diameter significantly increased from rest to exercise in both conditions. There were no differences (*P* > 0.05) between NAC and PLA. NAC, *N*‐acetylcysteine; pla, placebo.

**Figure 5:**
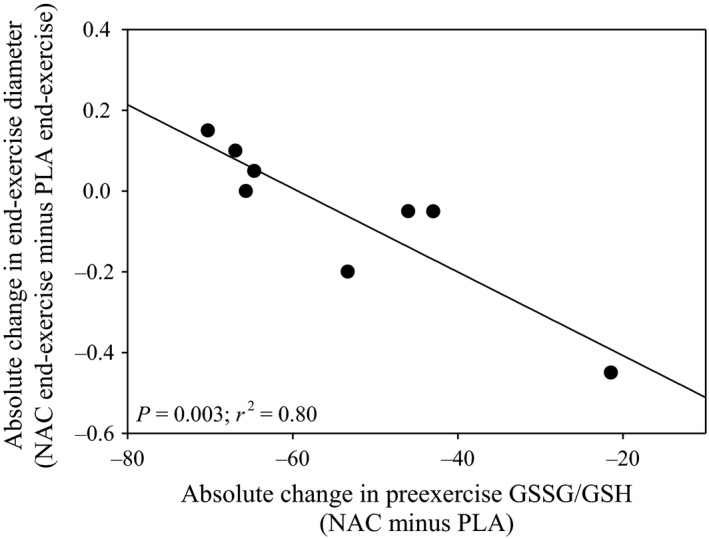
Relationship between preexercise GSSG/GSH and end‐exercise diameter. The relationship between absolute changes in preexercise GSSG/GSH (NAC minus PLA) and end‐exercise diameter (NAC minus PLA). There was a significant negative relationship (*r* ^*2*^ = 0.80) suggesting those with the greatest decrease in preexercise GSSG/GSH had larger end‐exercise diameter with NAC. NAC, *N*‐acetylcysteine; pla, placebo; GSSG, oxidized glutathione; GSH, glutathione.

Resting and exercising BABF for the NAC and PLA conditions are shown in Figure 3. Resting BABF was not different (*P* = 0.79) between NAC (99.3 ± 31.1 mL/min) and PLA (108.3 ± 46.0 mL/min) conditions. In both conditions, BABF significantly increased (*F* = 50.4, *P* < 0.001) from resting values; however, there were no differences (*F* = 0.74, *P* = 0.42) between NAC and PLA during exercise. End‐exercise BABF was not significantly different (*P* = 0.29) between NAC (413 ± 109 mL/min) and PLA (445 ± 147 mL/min). There was considerable intersubject variability in end‐exercise BABF with five of eight subjects having lower end‐exercise BABF with NAC compared to PLA. However, overall there was no relationship (*P* = 0.11) between the absolute change in end‐exercise BABF and exercise performance with NAC. The absolute change in BABF from rest to end‐exercise was not different (*P* = 0.49) between conditions. The end‐exercise blood flow (NAC: 390 ± 106 mL/min; PLA: 364 ± 44 mL/min; *P* = 0.60) and absolute change in blood flow (NAC: 299 ± 99 mL/min; PLA: 282 ± 32 mL/min; *P* = 0.72) were not different between NAC and PLA for the subjects (*n* = 5) demonstrating improved exercise performance with NAC. Figure 4 shows brachial artery diameters at rest and during handgrip exercise for both conditions. Resting brachial artery diameter was not significantly different (*P* = 0.52) between NAC (4.2 ± 0.6 mm) and PLA (4.2 ± 0.8 mm). Brachial artery diameter increased (*F* = 64.3, *P* < 0.001) from rest to exercise, but there were no differences (*F* = 0.39, *P* = 0.55) between NAC and PLA. The absolute change in brachial artery diameters from rest to end‐exercise was not significantly different (*P* = 1.0) between conditions. For the subjects (*n* = 5) who improved performance with NAC, the end‐exercise diameter (NAC: 4.3 ± 0.4 mm; PLA: 4.3 ± 0.4 mm; *P* = 1.00) and absolute change in diameter (NAC: 0.4 ± 0.1 mm; PLA: 0.5 ± 0.1 mm; *P* = 0.31) were not different between NAC and PLA. There were negative relationships between the absolute change in preexercise GSH/GSSG (NAC minus PLA) and the absolute change (NAC minus PLA) in resting (*r* ^*2 *^= 0.62; *P* = 0.02) and end‐exercise diameter (*r* ^*2 *^= 0.80; *P* = 0.003) (Fig. 5). No other significant associations were present between changes in diameter and plasma redox balance markers. Brachial artery shear rate increased (*F* = 51.6, *P* < 0.001) from rest to exercise for NAC and PLA, but no differences (*F* = 0.11, *P* = 0.75) occurred between groups. The absolute change in brachial artery shear rate from rest to end‐exercise was not different (*P* = 0.89) between conditions. No significant associations existed between the changes in plasma redox balance markers and blood flow or shear rate.

### NIRS

**Figure 6:**
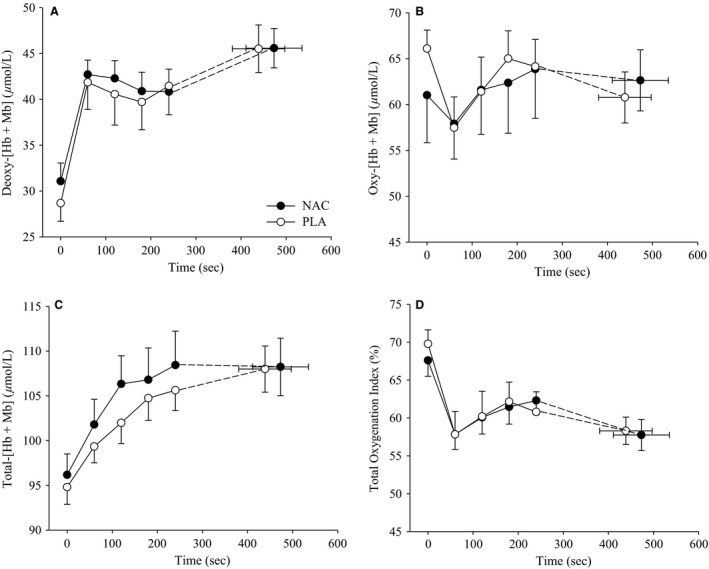
Mean NIRS muscle oxygenation during exercise with NAC and PLA. Mean deoxy‐[Hb + Mb] (A), oxy‐[Hb + Mb] (B), total‐[Hb + Mb] (C), and tissue oxygenation index (TOI) (D) for NAC (closed circles) and PLA (open circles) at rest and during exercise. There were no significant differences between NAC and PLA supplementation at rest or during exercise for any of the responses. NAC, *N*‐acetylcysteine; pla, placebo; deoxy‐[Hb + Mb], Deoxy‐[hemoglobin+myoglobin]; oxy‐[Hb + Mb], Oxygenated‐[hemoglobin+myoglobin].

The NIRS measurements, deoxy‐[Hb + Mb], oxy‐[Hb + Mb], total‐[Hb + Mb], and TOI, are shown at rest and during handgrip exercise in Figure 6. Resting deoxy‐[Hb + Mb] was not different (*P* = 0.42) at rest between NAC and PLA. Deoxy‐[Hb + Mb] significantly increased (*F* = 13.7, *P* < 0.001) during exercise for both NAC and PLA, but no significant differences (*F* = 0.13, *P* = 0.73) existed between conditions. There were no relationships (*P* > 0.05) between the absolute change in end‐exercise deoxy‐[Hb + Mb] and plasma redox balance markers. Oxy‐[Hb + Mb] was not different (*F* = 0.058, *P* = 0.82) at rest or during exercise between NAC and PLA. At rest, total‐[Hb + Mb] was not significantly different (*P* = 0.70) at rest between PLA and NAC. Total‐[Hb + Mb] increased (*F* = 12.2, *P* < 0.001) with exercise in both conditions, but no differences (*F* = 0.47, *P* = 0.52) existed between NAC and PLA. TOI was not different at rest or during exercise between NAC and PLA (F = 0.028, *P* = 0.87). The end‐exercise deoxy‐[Hb + Mb] (NAC: 49.3 ± 2.0 *μ*mol/L; PLA: 49.7 ± 6.1 *μ*mol/L; *P* = 0.91) and total‐[Hb + Mb] (NAC: 106.8 ± 7.1 mL/min *μ*mol/L; PLA: 109.4 ± 7.8 *μ*mol/L; *P* = 0.64) were not different between NAC and PLA for the subjects (*n* = 5) who improved exercise performance with NAC.

## Discussion

### Major findings

The major findings of this study are that an acute oral supplementation of NAC does not affect blood flow or muscle oxygenation characteristics at rest or during severe‐intensity handgrip exercise in healthy men. NAC supplementation did lead to increases in preexercise plasma CySH, tCyS, and CySH:tCyS. Lastly, handgrip exercise performance was not improved with an acute dose of NAC supplementation.

### NAC supplementation

Previous studies have used both intravenous and oral NAC supplementation to modify plasma redox balance (Ferreira and Reid 2008). Recently, oral NAC ingestion has been utilized because it is a more practical approach for common usage because intravenous infusion is only approved by the Food and Drug Administration for acetaminophen overdose treatment (Ferreira et al. 2011). Chronic (3–9 days) and acute oral NAC supplementation has been shown to alter plasma redox balance in healthy subjects (Ferreira et al. 2011; Corn and Barstow 2011; Matuszczak et al. 2005; Slattery et al. 2014); however, this is not a consistent finding (Kelly et al. 2009; Trewin et al. 2013). Ferreira et al. (2011) recently reported that 70 mg/kg was the highest oral acute dosage that could be well‐tolerated by subjects without adverse reactions. This dosage has been reported to increase tGSH (Corn and Barstow 2011) as well as increase CySH, tCyS, CySH:tCyS, and decrease GSSG (Ferreira et al. 2011). The changes in tGSH, CySH, tCys, and CySH:tCyS in this study are in agreement with these previous studies. Interestingly, we observed an elevated GSSG with the acute NAC supplementation, which is in contrast to the previous study measuring GSSG following an acute oral NAC supplementation (Ferreira et al. 2011). This discrepancy may be due to differences in the method of delivery (capsule versus drinking solution).

### NAC and peripheral hemodynamics

In health, neither intravenous infusion nor oral supplementation of NAC has led to changes in resting blood flow (Smith et al. 2014; Nyberg et al. 2012). Our results are in agreement, and in addition show that resting muscle oxygenation characteristics are not altered with NAC supplementation in healthy men.

During strenuous exercise, the endogenous antioxidant system is unable to completely buffer the oxidant concentration leading to an accumulation of free radicals (Sen 1995). In regards to oxygen transport, free radicals and thiol oxidation can decrease nitric oxide production (Jaimes et al. 2001), negatively affect endothelial nitric oxide synthase (Huang et al. 2001), and lead to increased protein S‐glutathionylation (Chen et al. 2010, 2011); however, it remains unknown if this occurs during severe‐intensity exercise in young men. Previous studies have suggested that because NAC is an antioxidant and thiol donor it may improve blood flow during fatiguing exercise (Reid et al. 1994; Slattery et al. 2014; Reid 2015). In contrast, NAC infusion did not lead to increased plasma nitrite concentrations during severe‐intensity cycling exercise in young men (Bailey et al. 2011). Furthermore, NAC infusion did not alter blood flow during moderate‐intensity exercise (Nyberg et al. 2012) despite reducing venous GSSG in healthy men (Nyberg et al. 2014). Previous studies have shown that exercising blood flow is not different (Richardson et al. 2007; Kirby et al. 2009) or decreased (Copp et al. 2009) with antioxidant supplementation in health. Our findings that NAC did not modify blood flow or brachial artery vasodilation during severe‐intensity exercise are in agreement with these latter studies. Although NAC led to increases in plasma CySH, it is possible that the men in this study had adequate endogenous enzymatic and nonenzymatic antioxidant systems where NAC supplementation did not lead to augmented free radical scavenging; however, without postexercise plasma redox measurements this is unknown in this study. Interestingly, we observed a negative relationship between the absolute change in preexercise GSH/GSSG and the absolute change in resting and end‐exercise diameter between conditions. This finding is supported by previous studies showing an attenuated exercise‐induced vasodilation (Richardson et al. 2007) and vascular conductance (Copp et al. 2009) with antioxidant supplementation in health; however, further investigation of this finding is warranted incorporating pre and postexercise redox balance markers.

An additional primary purpose of this study was to determine if NAC affected muscle oxygenation characteristics during severe‐intensity exercise. We found that NAC did not alter muscle oxygenation characteristics during exercise. Recently, it has been shown that antioxidant supplementation decreases the O_2_ delivery‐O_2_ utilization balance in rats (Copp et al. 2009). It is likely that a population with greater oxidative stress may exhibit augmented blood flow and vasodilation with NAC as seen with antioxidant treatment with aging (Jablonski et al. 2007; Kirby et al. 2009).

### Exercise performance

Infusion and oral supplementation of NAC has been reported to delay fatigue in both small muscle mass (Reid et al. 1994; Supinski et al. 1997; Travaline et al. 1997; Matuszczak et al. 2005; Kelly et al. 2009), and whole‐body exercise (Corn and Barstow 2011; Medved et al. 2004b; McKenna et al. 2006; Slattery et al. 2014; Cobley et al. 2011). However, this is not a consistent finding in large muscle mass exercise possibly due to the training status, exercise intensity, and/or method of delivery (Medved et al. 2003, 2004a; Bailey et al. 2011; Trewin et al. 2013). In addition, it has been suggested that NAC supplementation may be most effective during submaximal exercise (rather than maximal) (Matuszczak et al. 2005; Reid et al. 1994; Ferreira and Reid 2008) and with small muscle mass exercise (Ferreira and Reid 2008). Unexpectedly, we did not observe improvements in exercise performance in small muscle mass exercise with NAC due to high intersubject variability. However, consistent with previous studies (Bailey et al. 2011; Medved et al. 2004a), we found a variable response with most subjects (5 of 8) improving exercise performance with NAC supplementation. For whole‐body exercise performance, NAC appears to be more effective in endurance‐trained subjects (Medved et al. 2004a,b), but the role of training status during small muscle mass exercise is unknown. Recently, Ferreira et al. (2011) using the same dosage reported a relationship between exercise performance and preexercise CySH:tCyS levels. Interestingly, we did not observe any relationships between preexercise plasma redox balance values and exercise performance.

### Limitations

Several limitations may have influenced our results. First, the ultrasound used in this study does not allow for simultaneous measurements of velocity and diameter. Therefore, the timing of the image capture occurred during diastole and one vessel diameter was used at each time point to calculate blood flow (Ade et al. 2012; Broxterman et al. 2014; Smith et al. 2014). Second, postexercise plasma redox balance measurements would have provided valuable insight regarding the extent to which NAC altered redox status during exercise compared to PLA, as well as the adequacy of reactive oxygen species scavenging during exercise.

## Conclusion

In conclusion, we found that an acute oral supplementation of NAC did not modify the blood flow or vasodilatory response during severe‐intensity small muscle mass exercise. In addition, we did not observe differences in muscle oxygenation characteristics at rest or during exercise with NAC. Future studies should examine if blood flow and vasodilation is improved with NAC in populations with greater oxidative stress.

## Conflict of Interests

None declared.
